# Systemic reactivation of allergic contact dermatitis: cosmetics may be the principal felons but foods could be accessory to the crime

**DOI:** 10.1186/2045-7022-5-S1-O16

**Published:** 2015-03-11

**Authors:** Jadwiga Kalicinska, Radoslaw Spiewak

**Affiliations:** 1 Jagiellonian University, Dept of Experimental Dermatology and Cosmetology, Krakow, Poland

## Background

There are sensitizing haptens that are used as ingredients of both cosmetic products and foods. This provokes the question whether people with contact allergy to cosmetics could also develop dermatitis following systemic exposure to the provoking haptens present in foods - a phenomenon referred to as "systemic reactivation of allergic contact dermatitis" (SRACD).

## Method

Ingredients declared on packages of 150 cosmetics and 150 food products available in chain superstores were analyzed. The components identified in both cosmetics and foods were further analysed for their sensitizing potential based on available epidemiological and experimental data.

## Results

There were 9 haptens with known sensitizing potential occurring both in food and cosmetics - at least one of them was present in 65% food products and 87% cosmetics analyzed (Table). These sensitizers common to cosmetics and foods were predominantly present in bath lotions, shower gels, as well as filled chocolates, mayonnaise and fruit nectars.

## Conclusion

Cosmetics and food products share a range of ingredients known as causes of delayed type allergy. The possibility of systemic reactivation of allergic contact dermatitis by ingesting food products containing the same haptens as cosmetics should be considered in patients with confirmed contact allergy to cosmetic ingredients, who also demonstrate a disseminated, flexural or intertriginous pattern of eczema.

